# Spontaneous Detachment of the Leading Head Contributes to Myosin VI Backward Steps

**DOI:** 10.1371/journal.pone.0058912

**Published:** 2013-03-18

**Authors:** Keigo Ikezaki, Tomotaka Komori, Toshio Yanagida

**Affiliations:** 1 Graduate School of Frontier Biosciences, Osaka University, Suita, Osaka, Japan; 2 Quantitative Biology Center (QBiC), RIKEN, Suita, Osaka, Japan; 3 Center for Information and Neural Networks (CiNet), Suita, Osaka, Japan; 4 Immunology Frontier Research Center (IFReC), Osaka University, Suita, Osaka, Japan; Consejo Superior de Investigaciones Cientificas, Spain

## Abstract

Myosin VI is an ATP driven molecular motor that normally takes forward and processive steps on actin filaments, but also on occasion stochastic backward steps. While a number of models have attempted to explain the backwards steps, none offer an acceptable mechanism for their existence. We therefore performed single molecule imaging of myosin VI and calculated the stepping rates of forward and backward steps at the single molecule level. The forward stepping rate was proportional to the ATP concentration, whereas the backward stepping rate was independent. Using these data, we proposed that spontaneous detachment of the leading head is uncoupled from ATP binding and is responsible for the backward steps of myosin VI.

## Introduction

Myosin VI is an ATPase motor protein responsible for many cellular functions including endocytosis, protein secretion, and the maintenance of both the Golgi morphology and stereocilia [1]. Recently, we proposed that myosin VI moves following three types of steps: large and small forward steps (minus end directed), and backward steps (plus end directed) [2]. A number of models have explained that the forward steps emerge from an ADP-release gating [3], [4] or ATP-binding gating mechanism [5] that acts on the leading head in combination with a preferential binding mechanism that acts on the detached trailing head [6], [7], [8]. However, all these models fail to provide a satisfactory explanation for backward steps. According to the preferentially binding mechanism, stochastic ADP-release from the leading head results in ATP-binding and detachment. The now detached leading head should then preferentially bind to the binding site from which it detached resulting in no displacement. Yet we have shown using high-speed, gold nano-particle dark field imaging that this head can bind to the backward site to create a backward step [2]. In fact, we never observed binding to the original position.

In this study, to clarify how myosin VI generates its backward steps, we analyzed ATP-dependent stepping rate changes and step-type ratio changes using FIONA [9]. Our results indicate that spontaneous detachment of the leading head contributes to backward steps, meaning ATP-binding does not couple with myosin-VI backward steps.

## Results

### Apparent ATP-dependent stepping rates and probability of myosin VI steps

**Figure 1:**
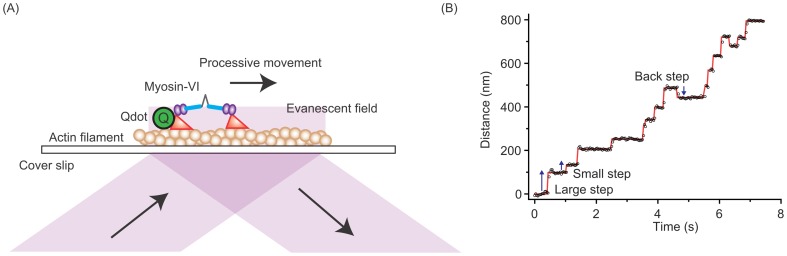
Experimental system for single molecule measurements of myosin VI. (A) A Qdot585-labeled myosin VI moving on an actin filament was illuminated using an evanescent field. Myosin VI was biotinylated via HaloTag at its N-terminus (motor domain) using biotin-Halo-ligand. A streptavidin conjugated Qdot585 (Life technologies) was attached to the motor domain of myosin VI using avidin-biotin interactions. (B) A typical stepping trace of myosin VI at 200 µM ATP. Examples of large, small and backward steps are indicated by the arrows.

**Figure 2:**
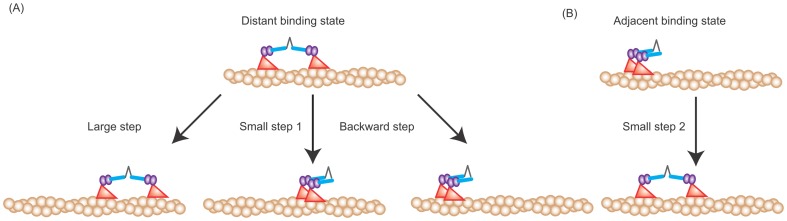
Correlation between myosin VI step types. (A) Steps from the distant binding state. A large step occurs only from the distant binding state and results in the distant binding state; small steps 1 from the distant binding state result in the adjacent binding state; backward steps only occur from the distant binding state and result in the adjacent binding state. (B) Steps from the adjacent binding state. From the adjacent binding state, myosin VI can only take a small step. Here, we should note an apparent correlation between steps (e.g. a large step following a backward step and a large step or backward step following a small step 1), which is inconsistent with our models, appears in the example traces (Fig. 1B and Fig. S1). The inconsistency is because which head is stepping from the adjacent binding state is unknown [11]. We have previously shown that either head has an equal probability of taking the next step from the adjacent binding state [11]. Our results here likely undercount the number of small steps by the labeled head, but at the same time equally overcount the number of steps following the adjacent binding state made by the other head because of our labeling method. Had we labeled both heads in our experiments, we would expect to see our model satisfied [2].

To clarify how the ATP hydrolysis cycle relates to myosin VI forward and backward steps, we analyzed stepping rates by single molecule imaging. Myosin VI labeled with Qdot585 had their processive movement visualized by total internal reflection fluorescence microscopy (Fig. 1A), and their steps tracked by FIONA [9] (Fig. 1B, Fig. S1). As a result, we observed two actomyosin binding states [2]: a distant binding state, in which the inter-head distance is 36 nm, a value consistent with the half helical pitch of an actin filament, and an adjacent binding state, in which the inter-head distance is less than 10 nm (Fig. 2).

**Figure 3:**
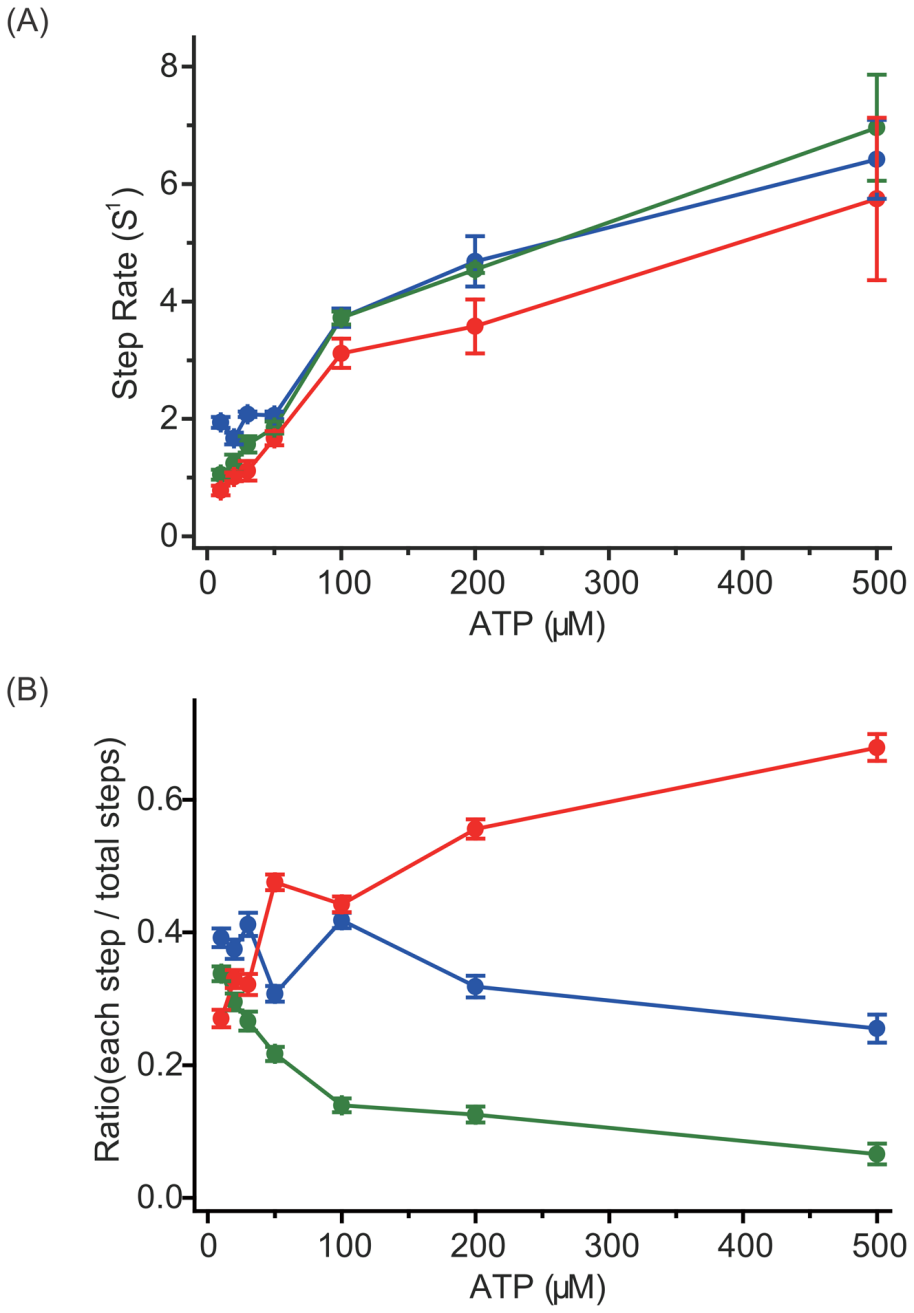
The stepping rate and the probability of step type at various ATP concentrations. (A) Stepping rate of each step type at various ATP concentrations (red circles, large steps; blue circles, small steps; green circles, backward steps). The stepping rate for large and small steps were calculated by fitting the dwell time distribution using a convolution of two exponentials (tk^2^ exp (-kt)) (Fig. S2A, B), while that of backward steps was calculated by fitting the dwell time distribution using a single exponential decay function (Fig. S2C). (B) Probability of step types at various ATP concentrations (red circles, large steps; blue circles, small steps; green circles, backward step). The probabilities were calculated by fitting the stepping size distribution (Fig. S2D) with a three-Gaussian function. All fits were performed using Origin 7.5 (OriginLab).

According to the dwell time distributions (Fig. S2), the apparent stepping rate for each step type can be estimated. Assuming that a forward step is due to the trailing head binding forward and a backward step by the leading head binding backward, the apparent stepping rates of all three step types can be described as *k_f_*+*k_b_*, where *k_f_* and *k_b_* describe the actual forward and backward stepping rates, respectively. The apparent ATP-dependent stepping rates of all three step types were measured at 10–500 µM ATP (Fig. 3A). Although the apparent stepping rates were found similar, we could not make the same conclusion about the actual stepping rates. Because myosin VI is a dimer, the two heads undergo a competing process to take the next step. The apparent stepping rates describe such a process, whereas the actual stepping rates describe a non-competing process. Therefore, to clarify how myosin VI generates backward steps, the actual stepping rates for forward and backward steps, which can be determined from the apparent stepping rates, are required (see below).

Regarding the probability of the step types, the proportion of large forward steps decreased with decreasing ATP, while the proportion of the other two increased (Fig. 3B). The probability of each step was calculated by fitting the stepping size distribution (Fig. S2D) with a three-Gaussian function.

### Analysis of stepping rates and step-type probabilities

Because we have previously reported that the type of forward steps taken from the distant binding state is determined by the structural state of the leading head's lever arm (i.e. the pre-power or post-power stroke state) when the leading is bound and the trailing head is detached [2], we define the probability of a small forward step as *s*. Lastly, the apparent stepping rate from the distant binding state, either through a backward or a forward step, is *k_app_* = *k_f_*+*k_b_*. According to the relationship between the rate constants of competing reactions (see Materials and Methods) [10], the ratio of the three step types from the distant binding state can be described as large (*r_L_distant_*): small (*r_s_distant_*): backward (*r_B_distant_*)  =  (1−*s*)*k_f_*: *sk_f_*: *k_b_*.

From the adjacent binding state myosin VI will always take a small forward step [2], resulting in the distant binding state (Fig. 2B). If from the distant binding state a small forward or backward step occurs, then myosin VI will take the adjacent binding state (Fig. 2A). Large forward steps and backward steps can occur only from the distant binding state (Fig. 2A). We found that the likelihood of myosin VI taking the distant binding or adjacent binding state at the start of processive movement (61% and 39%, respectively; n = 54) equaled that at the termination of movement (61% and 39%, respectively; n = 61) (Fig. S3). This property allowed us to combine the number of steps from several traces when doing our analysis. Here, we defined the distant binding state as that with an inter-head distance of over 30 nm and the adjacent binding state as that under 15 nm. The frequency of small forward steps following small forward or backward steps from the distant binding state, *sk_f_*+*k_b_*, was added to *r_s_distant_* to calculate the probability of the total number of small forward steps, resulting in *r_s_* = 2*sk_f_*+*k_b_*, where *r_s_* equals the number of total small forward steps. The probabilities of large steps (*r_L_*) and backward steps (*r_B_*) equaled *r_L_distant_* and *r_B_distant_*, respectively, since these steps can occur only from the distant binding state. Therefore, *r_L_*: *r_s_*: *r_B_* can be rewritten as (1−*s*)*k_f:_* (2*sk_f_*+*k_b_*): *k_b_*, which means *s* can be described as:
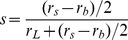
(1)


**Figure 4:**
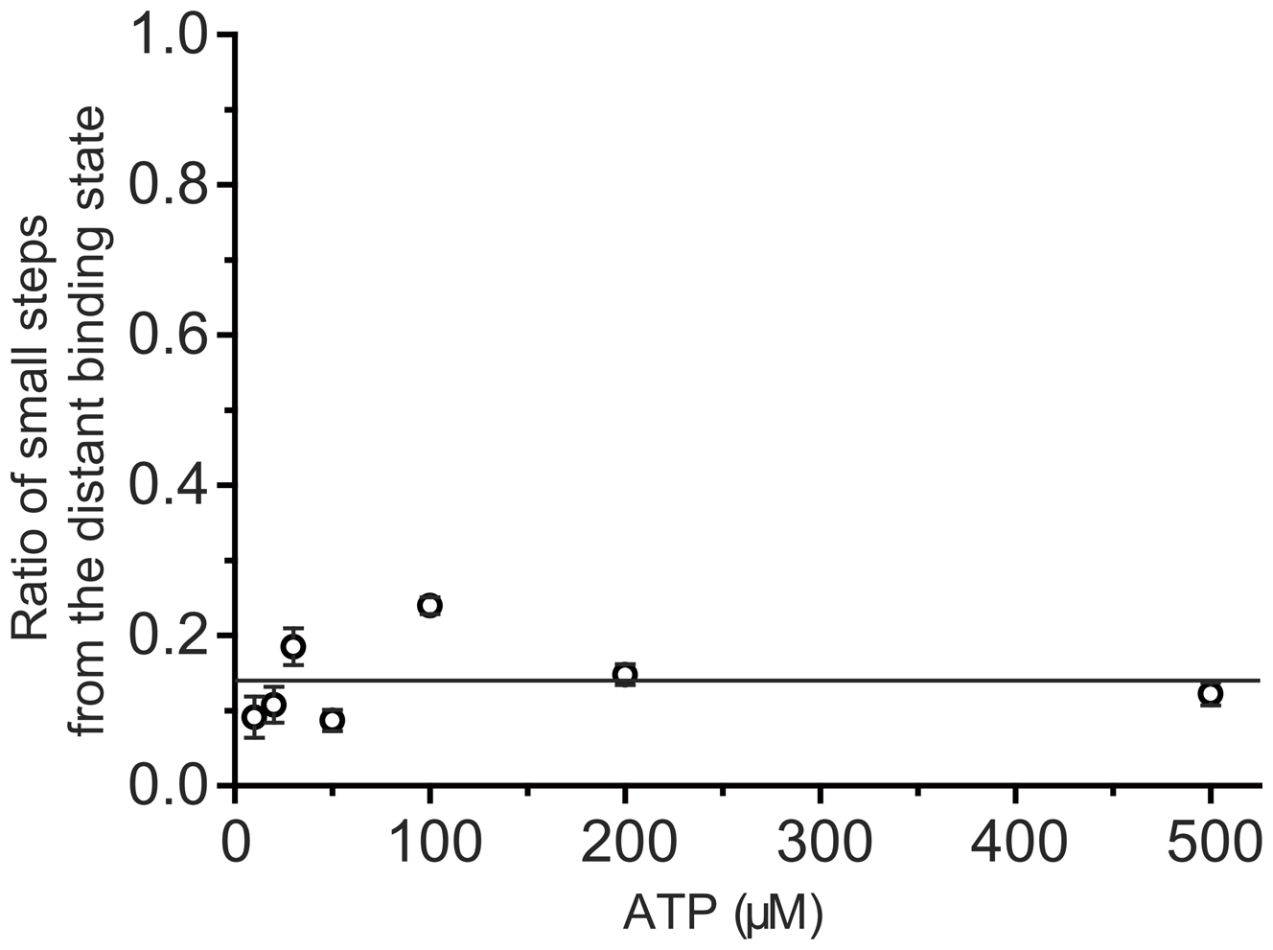
Calculation of small step frequency from the distant binding state. The distribution of small steps, *s*, from the distant binding state was constant (0.14) against ATP concentration. Errors for the frequency were calculated using errors for each apparent stepping rate, the law of error propagation and the freeware Maxima.

*s* was found independent of ATP concentration and equaled 0.14±0.021 (Fig. 4).

When myosin VI is in the adjacent binding state, the terms ‘leading head’ and ‘trailing head’ no longer apply, as we have shown that either head has an equal probability of taking the next step [11]. Because of this equal probability, our results likely undercount the number of small steps for one head, which is consistent with half the number of small step 2 from the adjacent binding state, (*sk_f_*+*k_b_*)/2, but at the same time equally overcount the number of steps following the adjacent binding state made by the other head, (*sk_f_*+*k_b_*)/2, meaning which head takes the ensuing step from the adjacent binding state is unlikely to compromise our results.

### Estimation of actual stepping rate constants from observed rate constants

That *r_L_*: *r_B_*  =  (1−*s*)*k_f_*: *k_b_* means *k_f_*: *k_b_*  =  *r_L_*/(1−*s*): *r_B_*. Thus, the actual forward and backward stepping rates can be estimated as follows.
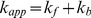
(2)

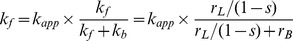
(3)

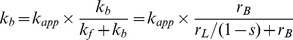
(4)


**Figure 5:**
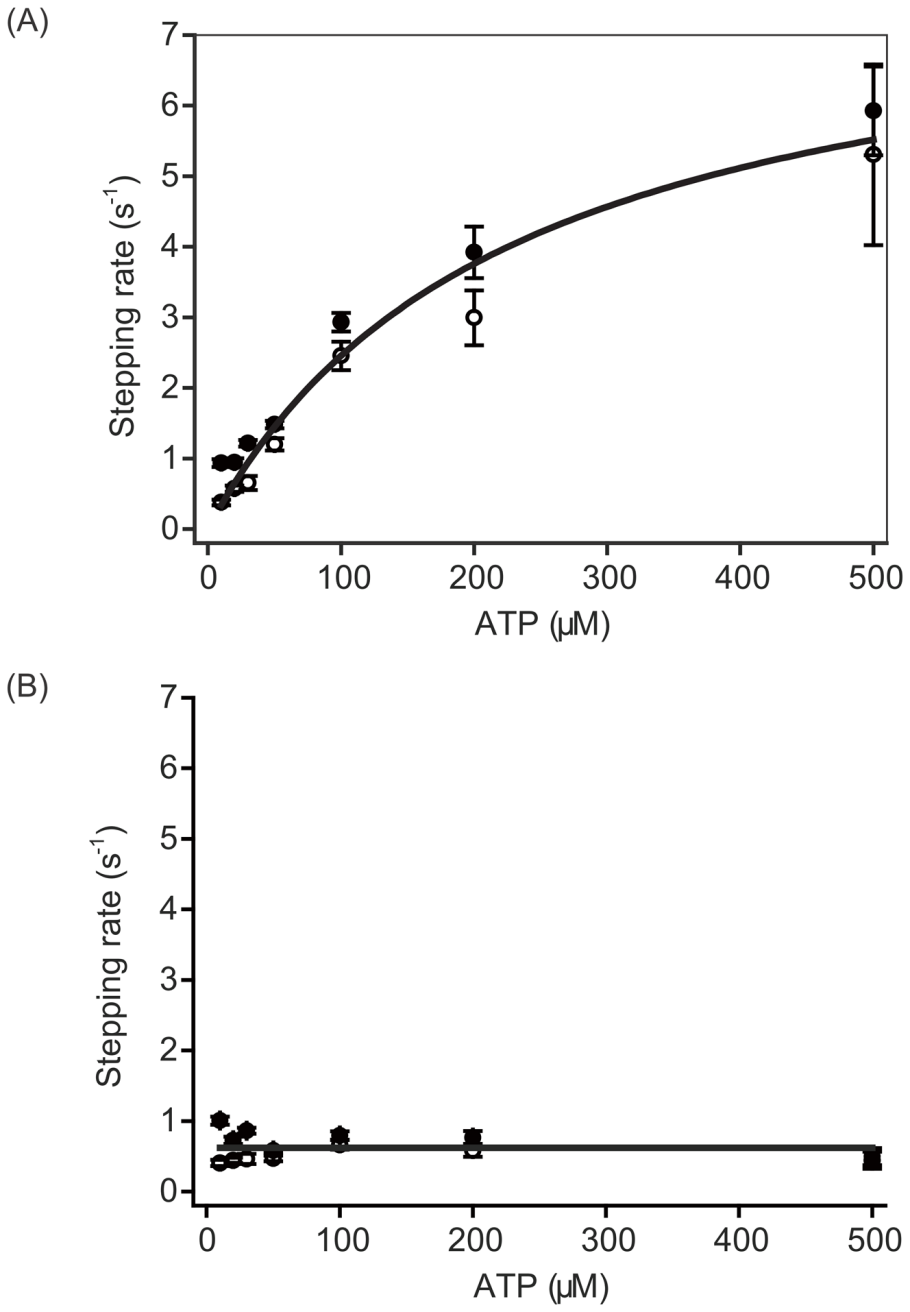
Actual stepping rate calculated from the apparent stepping rate and the step ratio. (A) Actual forward stepping rate. The distribution was well fitted to the function 1/k_f_ = 1/(k_ATP_[ATP])+1/k_ADP_ with k_ATP_ = 0.035 µM·s^−1^ and k_ADP_ = 8 s^−1^. (B) Actual backward stepping rate. The distribution had a constant value of 0.62, meaning that ATP-binding does not couple with backward steps. Open circles indicate values estimated from the apparent large step rate; closed circles indicate those from the apparent backward step rate. The errors of the actual forward and backward stepping rates were calculated using the error for each apparent stepping rate, the law of the error propagation and the freeware Maxima.

Therefore, according to equations 2–4 the actual forward stepping rate (Fig. 5A) and backward stepping rate (Fig. 5B) can be calculated from the apparent stepping rate (Fig. 3A) and the probabilities of the step types (Fig. 3B). The rate limiting states of the myosin VI ATP hydrolysis cycle at low ATP concentration had an ATP binding waiting time of 1/*k_ATP_*[ATP] and an ADP-release waiting time of 1/*k_ADP_* [12]. Thus, the actual forward stepping rate of *k_f_* was well fitted to the function 1/*k_f_*  = 1/*k_ATP_*[ATP]+1/*k_ADP_* with a *k_ATP_* of 0.035 µM•s^−1^ and *k_ADP_* of 8 s^−1^ (Fig. 5A), values that are consistent with biochemical experiments [12]. The actual backward stepping rate of *k_b_* was 0.62 s^−1^ (Fig. 5B). Should the distribution of *k_b_* be forced to fit the ATP binding coupled equation of 1/*k_b_* = 1/*k_ATP_*[ATP]+1/*k_ADP_*, then *k_ATP_* and *k_ADP_* would equal 180 µM•s^−1^ and 0.60 s^−1^, respectively. The ATP-binding rate of the leading head slows with intramolecular strain as *k_ATP_* = *k_0,ATP_*×exp (*Fδ*/*k_B_T*), where *k_0,ATP_* = 0.05 µM•s^−1^ and *δ* = 4 nm [5]. Assuming a backward force of 1 pN, which mimics the strain felt on the leading head, the above relationship for backward steps would result in *k_ATP_* = 0.02 µM•s^−1^, a value in complete disagreement with biochemical results, which suggests instead that ATP-binding does not couple with myosin-VI backward steps.

A spontaneous detachment of the myosin V leading head at about 1 s^−1^ has been reported previously [13]. A separate study reported that when myosin V is pulled to the backward direction at a super stall force (5 to 10 pN), it shows ATP-binding uncoupled backward steps because the leading head has a lower actomyosin binding force than that of the rear head [14]. Our result of an ATP-binding uncoupled myosin VI backward step rate of 0.62 s^−1^ is consistent with myosin V, indicating that myosin VI too generates backward steps by spontaneous detachment of the leading head.

## Discussion

Here we show that ATP-binding is coupled only to myosin VI forward steps. The two prevailing models for myosin V steps, however, assume that all steps are done in an ATP-dependent manner [6], [7], [8] Our results disagree, as they indicate that backward steps are better explained by a spontaneous detachment mechanism that is ATP independent, as described below.

### Mechanism of myosin VI backward steps

In the strain sensor model, intramolecular backward strain to the detached trailing head promotes phosphate release from the motor domain, resulting in preferentially binding to the forward binding site because of the transition from a weak to strong actomyosin binding state [6]. This model is unlikely to apply to backward steps if they occur from spontaneous detachment, which would make them ATP independent, however, because the model demands any step requires phosphate release from the detached leading head.

**Figure 6:**
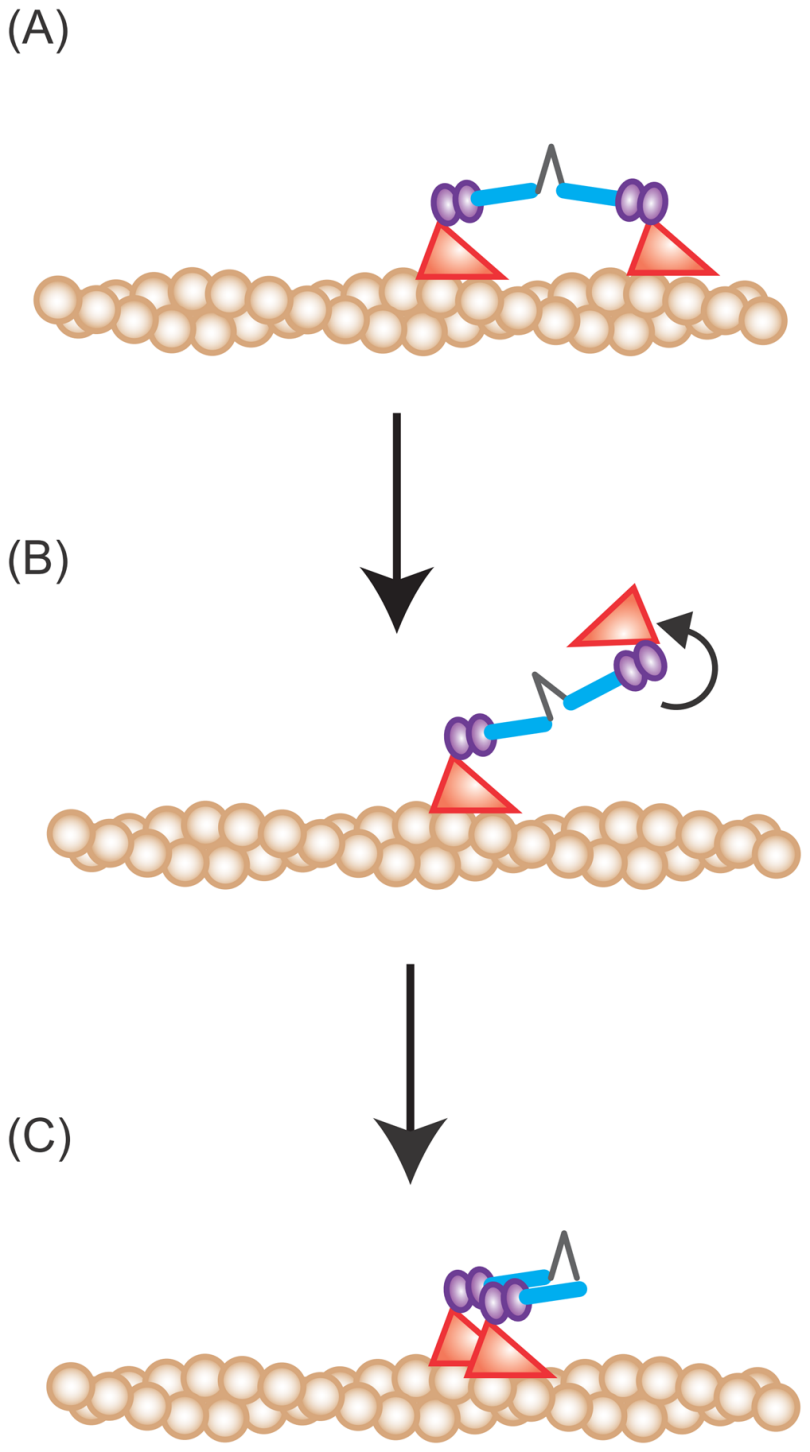
Model for backward steps. (A) Myosin VI backward steps occur only from the distant binding state. (B) The spontaneously detached leading head is in the ADP-bound or no nucleotide bound state. In either state, myosin VI takes the post-power stroke state, causing a motor domain rotation. (C) Upon binding, the head is in the pre-power stroke state and preferentially binds to the actin adjacent to the bound head, resulting in a backward step.

In the case of the toe up-down model, Shiroguchi *et al.* proposed that ATP-binding coupled with trailing head detachment induces a conformational change in the lever arm from the post-power stroke to pre-power stroke state, causing preferential binding to the forward binding site because of the geometry between the actin filament and the myosin head [7], [8]. Upon spontaneous detachment, the myosin VI head is in the ADP-binding or no nucleotide binding state (post-power stroke state) [11], [15]. This structure leads to preferential binding to the adjacent backward site (Fig. 6).

### Physiological meaning of spontaneous-detachment based backward steps

At cellular ATP concentrations (several mM) and no load, myosin VI performs unidirectional movement as a transporter and mainly takes forward steps. This is due to a higher ATP binding rate (Fig. 5A) to the trailing head than the 0.62 s^−1^ spontaneous detachment rate of the leading head. Therefore, at no load, the low frequency of backward steps should ensure myosin VI functions as a transporter for endocytosis [1].

Under high backward load, however, the forward stepping rate of the trailing head decreases [16] such that detachment of the leading head is enhanced [14]. This promotes myosin VI's anchoring function, which is important for stereocillia and Golgi complex maintenance [1], because of an increase in the frequency of backward steps from the distant binding state, which would result in a higher occurrence of the adjacent binding state.

At the same time, under backward load and in the adjacent binding state, the forward small stepping rate is sufficient for myosin VI to take the distant binding state. The result is a loop of small forward steps and backward steps, causing myosin VI to alternate between the adjacent binding and distant binding states. We recently reported that the tail domain of myosin VI, which is where myosin VI attaches to membrane structures via myosin VI binding proteins [17], does not move during a backward step or a small forward step from the adjacent binding state, and takes a structure that is particularly effective to resisting load and therefore ideal for anchoring. If it were the case that ATP-binding relates to both forward and backward steps, then at the high load condition all step rates from the distant binding state would decrease due to the backward strain on the heads. If so, there would be no change in the frequency of the adjacent binding state, compromising myosin VI's anchor function.

## Materials and Methods

### Relationship between rate constants of competing reactions and their probabilities

In the case the rate constant of an event does not change, the waiting time for the next event follows a single exponential decay function. Thus, the apparent distributions of the durations of competing reactions are described below with the rate constant *k*. 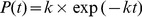
(5)

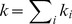
(6)


Here, the actual reaction rate of event *i* is described as *k_i_*, while the probability of that event is described as *k_i_*/*k* [10].

### Preparation of protein samples

To create dimeric myosin VI constructs, human myosin VI cDNA was truncated at Ala1021. This fragment included the motor domain, neck domain, and coiled-coil domain. To ensure myosin VI dimerization, the coiled-coil domain of the chicken gizzard smooth muscle myosin was appended at the Ala1021. For biotin labeling, a HaloTag (Promega) fragment was attached at the N- terminals. The sample contained His-tag at the C-terminus for protein purification. Protein purification was performed as previously described [2].

Qdot585 streptavidin conjugates and biotinylated myosin VI were incubated for single label imaging using FIONA [9].

### Microscopy observation and analysis

All experiments were performed according to previously reported methods [2]. Briefly, a 10 µl volume microchamber was made by placing a small coverslip (18×18 mm, No. 1 Thickness, Matsunami, Japan) over a larger one (22×32 mm, No. 1 Thickness, Matsunami) using double-sided adhesive tape (50 µm thickness). Next, 1.5 mg/ml actinin (Sigam-Aldrich) in assay buffer (AB: 20 mM HEPES-KOH (pH 7.8), 25 mM KCl, 5 mM MgCl_2_ and 1 mM EGTA) was adsorbed onto the glass surface, followed by a 3 min incubation, a 20 µl AB wash, and finally an injection of 2 µg/ml non-fluorescent phalloidin labeled actin filament solution in AB into the chamber. After another 3 min incubation and 20 µl AB wash, 5 mg/ml α-casein in AB was injected into the chamber. After a third 3 min incubation and 20 µl AB wash, MB (AB plus an oxygen scavenger system, ATP regeneration system and various ATP concentrations) mixed with Qdot labeled myosin VI was flowed into the chamber and the chamber was sealed with nail polish. Qdot conjugated myosin VI movement was imaged using total internal reflection fluorescence microscopy [18] and the corresponding fluorescent images were captured with an EMCCD camera (Andor iXon). The spot center for each frame was determined using a double Gaussian fit according to a published method [19], [20], [21] and analyzed. All steps were detected by eye.

## Supporting Information

Figure S1 **Myosin VI stepping traces at various ATP concentrations: 10 µM (A), 20 µM (B), 30 µM (C), 50 µM (D), 100 µM (E) and 500 µM (F).**
(TIF)

Figure S2 **Myosin VI dwell time distributions and step size distribution: (A) Histograms of dwell times just before forward large (60–150 nm) and (B) small (0–60 nm) steps.** The histograms were best fit by a convolution of two exponentials (tk^2^ exp (-kt)). The value of k for large and small steps was 3.6 s^−1^ and 4.5 s^−1^ at 200 µM ATP, respectively. (C) Histogram of dwell times just before backward steps. The histogram was best fit by a single exponential function with a rate constant of 4.7 s^−1^ at 200 µM ATP. (See Yildiz *et al.* [9] for details of the fitting functions). (D) Histogram of the distribution of myosin VI steps at 200 µM ATP. The distribution was fitted with a three Gaussian function with means ± standard deviation (S.D.) of 76 ± 7.8, 44 ± 11, and –41 ± 10 nm, and areas under the curve ± standard error of 5086 ± 133, 2911 ± 150, and 1148 ± 111. The black line indicates the sum of the three Gaussian functions. Although we could not distinguish which individual steps belonged to large and small steps in the overlapping region of the functions, we could estimate the probability of each event on the basis of the area of each peak (*r_L_*: *r_S_*: *r_B_*).
(TIF)

Figure S3 **The binding state of myosin VI at the start and end of processive movement.** At the start of processive movement (left), myosin VI takes the distant binding (61%) or adjacent binding states (39%) (n = 54). After myosin VI moves processively along an actin filament, it detaches to terminate processive moment (right). At this stage, the distant binding (61%) or adjacent binding states (39%) occur at the same frequency as the start of processive movement (n = 61). Here, we defined the distant binding state as when the inter-head distance is over 30 nm and the adjacent binding state as when it is under 15 nm. The inter-head distance was directly measured at the single molecule level using SHREC [11].
(TIF)
