# Supplementary figures and images for: Spontaneous Detachment of the Leading Head Contributes to Myosin VI Backward Steps
Source: PLoS One. 2013 Mar 18;8(3):e58912. doi: 10.1371/journal.pone.0058912 (PMC3601099; doi:10.1371/journal.pone.0058912)

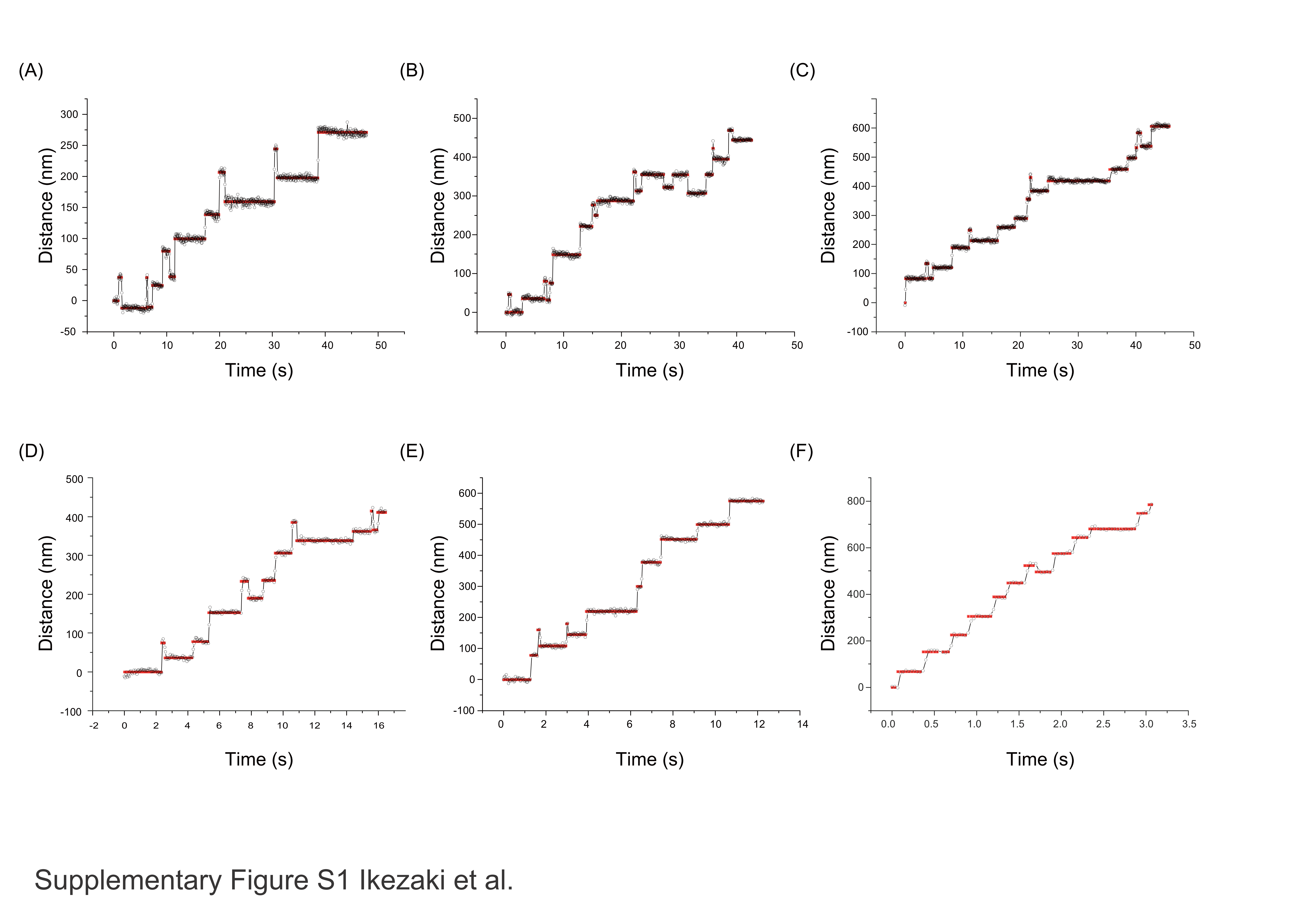

Supplement: Figure S1 — Myosin VI stepping traces at various ATP concentrations: 10 µM (A), 20 µM (B), 30 µM (C), 50 µM (D), 100 µM (E) and 500 µM (F). (TIF) [file pone.0058912.s001.tif]

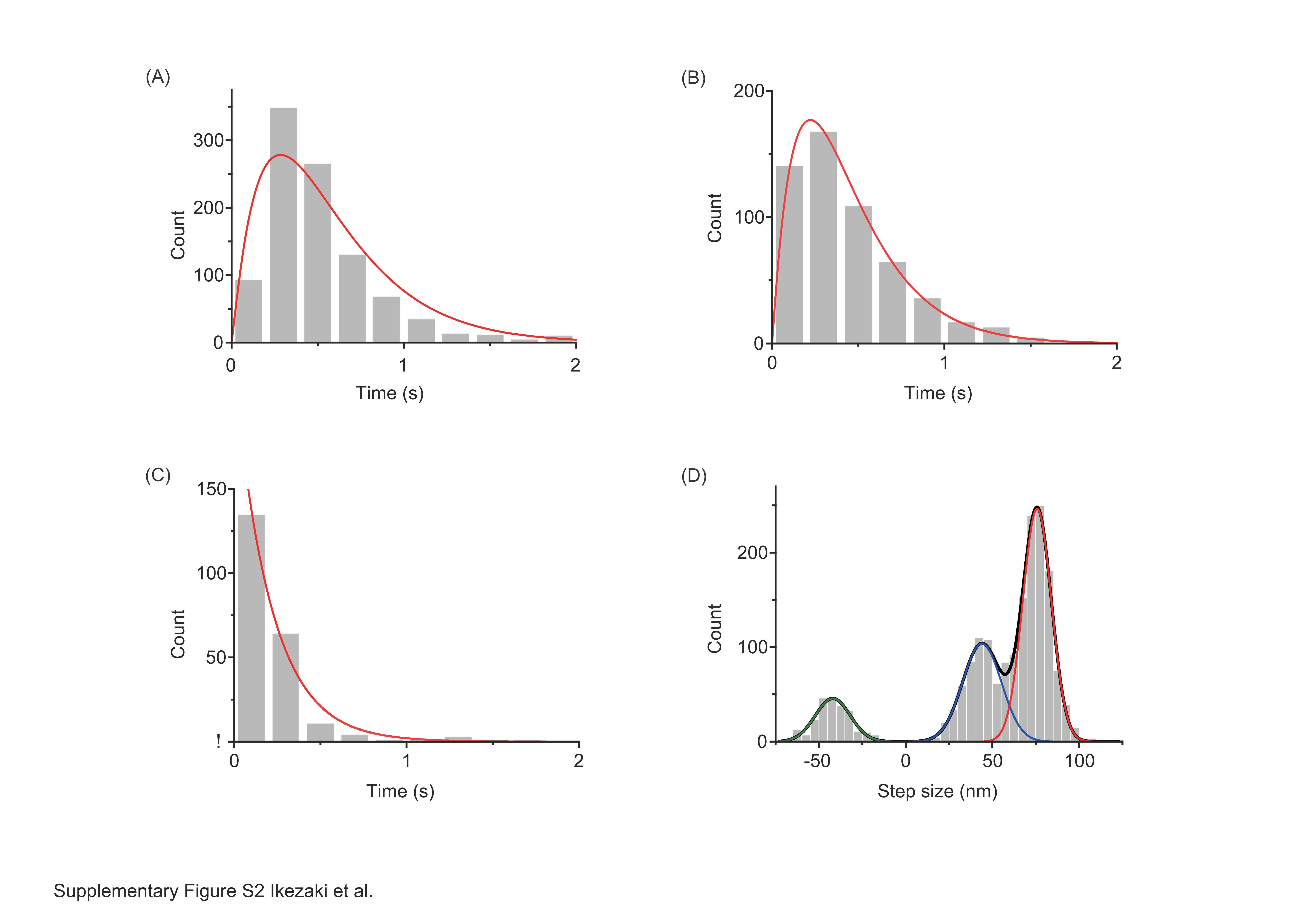

Supplement: Figure S2 — Myosin VI dwell time distributions and step size distribution: (A) Histograms of dwell times just before forward large (60–150 nm) and (B) small (0–60 nm) steps. The histograms were best fit by a convolution of two exponentials (tk2 exp (-kt)). The value of k for large and small steps was 3.6 s−1 and 4.5 s−1 at 200 µM ATP, respectively. (C) Histogram of dwell times just before backward steps. The histogram was best fit by a single exponential function with a rate constant of 4.7 s−1 at 200 µM ATP. (See Yildiz et al. [9] for details of the fitting functions). (D) Histogram of the distribution of myosin VI steps at 200 µM ATP. The distribution was fitted with a three Gaussian function with means ± standard deviation (S.D.) of 76 ± 7.8, 44 ± 11, and –41 ± 10 nm, and areas under the curve ± standard error of 5086 ± 133, 2911 ± 150, and 1148 ± 111. The black line indicates the sum of the three Gaussian functions. Although we could not distinguish which individual steps belonged to large and small steps in the overlapping region of the functions, we could estimate the probability of each event on the basis of the area of each peak (rL: rS: rB). (TIF) [file pone.0058912.s002.tif]

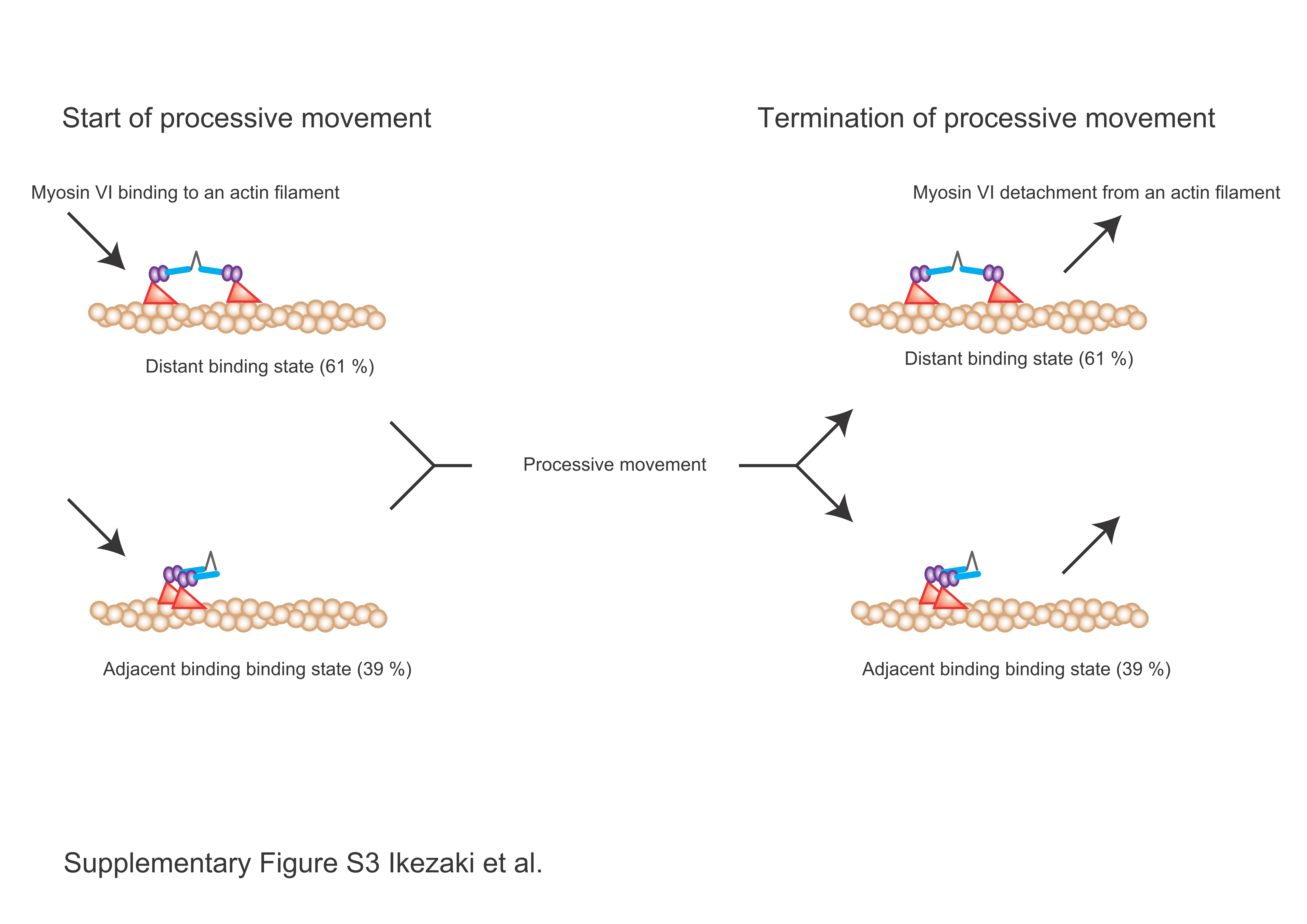

Supplement: Figure S3 — The binding state of myosin VI at the start and end of processive movement. At the start of processive movement (left), myosin VI takes the distant binding (61%) or adjacent binding states (39%) (n = 54). After myosin VI moves processively along an actin filament, it detaches to terminate processive moment (right). At this stage, the distant binding (61%) or adjacent binding states (39%) occur at the same frequency as the start of processive movement (n = 61). Here, we defined the distant binding state as when the inter-head distance is over 30 nm and the adjacent binding state as when it is under 15 nm. The inter-head distance was directly measured at the single molecule level using SHREC [11]. (TIF) [file pone.0058912.s003.tif]
